# Engaging nursing home residents in clinical research: insights from a patient advisory board, a patient advocate, and a study team

**DOI:** 10.1186/s40900-024-00648-1

**Published:** 2024-10-28

**Authors:** Simone Böbel, Ansgar Gerhardus, Carolin Herbon, Hannah Jilani, Kim Isabel Rathjen, Guido Schmiemann, Imke Schilling

**Affiliations:** 1 Department for Health Services Research, Institute of Public Health and Nursing Research, University of Bremen, Grazer Straße 4, 28359 Bremen, Germany; 2 Health Sciences Bremen, University of Bremen, 28359 Bremen, Germany; 3 Department of Health Ethics and Society, Care and Public Health Research Institute, Maastricht University, Maastricht, 6229 HA The Netherlands; 4 Kompetenzzentrum für Klinische Studien, Linzer Straße 4, 28359 Bremen, Deutschland

**Keywords:** Patient and public involvement, Nursing home residents, Nursing homes, Clinical Research, Polypharmacy, Patient Advisory boards, Patient advocate, PPI, PAB, PA

## Abstract

**Background:**

Patient and Public Involvement (PPI) is increasingly recognized as an essential aspect of clinical research, particularly for ensuring relevancy and impact of research to those most affected. This study addresses the gap in involving older patients, particularly nursing home residents, in the research process by exploring motivations, expectations, and experiences of nursing home residents in Bremen, Germany, involved in PPI for developing a clinical trial on polypharmacy.

**Methods:**

Two Patient Advisory Boards (PABs) were established in nursing homes as part of the INVOLVE-Clin project. A Patient Advocate (PA) facilitated communication between nursing home residents and researchers. A qualitative case study approach was employed, involving semi-structured interviews and group discussions with nursing home residents and researchers. Data was analyzed using structured qualitative content analysis.

**Results:**

The study found varied motivations and expectations between nursing home residents and researchers. Nursing home residents valued the social interaction and the opportunity to voice their health concerns, while researchers aimed to incorporate patients’ perspectives into study design. The PA was considered crucial in facilitating communication between nursing home residents and researchers. Challenges included the complexity of the study topic and the need for methodological adjustments to suit nursing home residents´ cognitive abilities. Generally, PAB participation was experienced to provide mental stimulation and increased confidence among nursing home residents in discussing their medication management. The PAB’s influence led to the decision not to conduct a polypharmacy study.

**Discussion:**

The findings underscore the importance of flexible approaches to PPI, particularly when involving older nursing home residents. Methodological adjustments, such as tailoring content and structure of PABs, and the inclusion of additional boards for diverse perspectives, are vital for effective involvement. The study also highlights the need for ongoing innovation in PPI methods to ensure meaningful engagement of older patients in clinical research.

**Conclusion:**

This study contributes essential insights into the practical implementation of PPI with nursing home residents, highlighting the need for patient-centric approaches that recognize their unique challenges and contributions. These findings are critical for shaping scientifically robust but also socially relevant and impactful research, especially in an aging society.

**Supplementary Information:**

The online version contains supplementary material available at 10.1186/s40900-024-00648-1.

## Abstract (plain language summary)

This study looked at how nursing home residents in two nursing homes in Bremen, Germany, feel about being part of health research. It focused on their reasons for joining, what they hoped to get out of it, and their experiences. The study used two groups, called Patient Advisory Boards (PABs), in these nursing homes. An important part of making this work was having someone called a Patient Advocate, who talked to the nursing home residents and communicated their perspectives to the research team. Researchers talked to the residents and other researchers through interviews and group discussions. The study found that nursing home residents and researchers had different reasons for being involved. The nursing home residents enjoyed the chance to talk with others and share their health worries, while the researchers wanted to make sure the study considered the views of older patients. There were some challenges for both, researchers and nursing home residents. The topic of the study, which was about using many different medicines (polypharmacy), was complex. Nursing home residents would need easier methods to take part. Nonetheless, being part of the PABs was experienced to be good by the nursing home residents. It helped them think more about their health and feel more confident talking about their medicines. The study shows that when including nursing home residents in health research, it’s important to think about their specific needs to ensure meaningful engagement.

**Supplementary Information:**

The online version contains supplementary material available at 10.1186/s40900-024-00648-1.

## Background

Patient and Public Involvement (PPI) in research enhances the relevance, quality, and applicability of research by aligning it with patients’ and/or publics’ needs by co-producing research collaboratively “with or by” the target group rather than “to, about, or for” them [1–3]. Users of healthcare services should have a say in research processes, translation into clinical practice, and broader healthcare system changes, as they are the ones most directly impacted by the resulting benefits and drawbacks [2, 4]. The significance of PPI in clinical research has gained recognition, and has become a requirement in funding applications, journal policies, national policies, and international programs [2, 5–10]. For PPI to be effective, it requires proper training for both patients and researchers, clear communication, supportive infrastructure, and ongoing evaluation and feedback [11–13].

Despite the growing push for greater patient involvement in research, certain marginalized groups, including older persons and care-dependent persons, are less likely to actively participate in clinical research [14]. This is often due to the perception that older patients are unable or unwilling to contribute to the planning and execution of clinical trials [15, 16]. It is true that older patients may require additional support in terms of logistics, time and costs to overcome auditory, visual, and cognitive limitations that could otherwise hinder their active involvement [14, 17–20]. However, this should not lead to the exclusion of groups that will be most affected by the research outcomes. Our previous literature review indicated that involving older people in research is feasible, but it poses numerous challenges (e.g. with regard to communication, timing and continuity, diversity, choice of location) [20]. The involvement must be designed to consider strategies that enhance effective involvement (e.g., visualization and accessible communication, building strong relationships, flexibility) and the capabilities, knowledge, and availability of the people involved [20]. Tokenistic involvement for the sake of meeting policy or grant requirements should be avoided. Instead setups should be created that enable meaningful patient involvement [11, 21].

With an aging society, research and its translation into healthcare practices must accurately reflect the priorities and needs of older patients and stakeholders. Consequently, PPI involving older patients in research is gaining significance, and methods for actively engaging older patients in a meaningful manner should be explored [22–24]. Different motivations, expectations, and disappointing experiences can often create barriers to understanding between older patients and researchers, which potentially hinder effective involvement [25, 26].

In the context of PPI in health system research, in Germany, even though some PPI networks, such as “Netzwerk Partizipative Gesundheitsforschung (PartNet)”, were founded almost twenty years ago, only in recent years have guidelines and policies on the necessity and methods of involvement been more prominently published. This indicates that Germany is lagging behind Anglo-Saxon countries in this area [13, 27–29].

### Research aim

Therefore, our aim was to examine the motivation, expectations, and experiences of involving older persons in a nursing home setting (hereafter referred to as nursing home residents) in the planning of a study through a patient advisory board. Nursing home residents, a subgroup of older persons, were invited for this study, as they represent a group that is frequently underrepresented in research [30], even though their perspective and experiences are crucial to ensure that research is relevant and meaningful to them. Yet, it is important to note that nursing home residents have unique needs and perspectives that may differ from those of older adults living independently.

To gain insights into the perspectives of stakeholders involved in PPI, we formulated the following research questions:

- What **motivates** nursing home residents and researchers to engage in PPI, specifically to become part of a patient advisory board (PAB) or study planning team (to develop a clinical trial on polypharmacy)?
- What **expectations** do nursing home residents and researchers have regarding their involvement in a PAB or study planning team (for the development of a clinical trial on polypharmacy)?
- What **experiences** did nursing home residents and researchers make with the PAB, study planning team and intermediary patient advocate (PA) to facilitate PPI (for the development of a clinical trial on polypharmacy)?

This research was conducted within the framework of the INVOLVE-Clin project (funded by BMBF: 01GL1726). The INVOLVE-Clin project explored the involvement of older patients in clinical research. Its’ aim was to develop a guideline on PPI with older patients.

## Methods

A qualitative prospective case study was conducted from 2019 to 2022 within two nursing homes in Bremen, Germany. A comprehensive project description can be found in the previously published study protocol [31]. Ethical approval for this study was granted by the ethics committee of the University of Bremen, Germany, on 16 October 2018.

Our study project received support from its inception by two persons over the age of 65, serving as consultants. One was a nursing home resident, while the other had experience with the nursing home environment through volunteer work. They regularly met with the researchers to discuss and plan this study by contributing to the establishment of the PABs, ensuring they were safe spaces for the participating nursing home residents. Throughout the study, their insights significantly enriched the study design and the discussion of results from the PABs within the study team.

To ensure transparency and comprehensive reporting, we adhered to the consolidated criteria for reporting qualitative research (COREQ) [32] and followed the GRIPP2 checklist for reporting patient involvement in research [33]. As patient and public involvement (PPI) may imply that other groups aside from patients were involved, the term patient involvement (PI) was used rather than PPI to bring awareness to our set-up.

### Setting: nursing homes in Germany

In Germany, three out of four persons in need of care are cared for at home by relatives or other volunteers and sometimes supported or fully cared for by ambulatory care services. When ambulatory care no longer suffices there are possibilities of part-time care in nursing homes (e.g. for several hours or overnight) or full-time care in nursing homes. Moreover, older persons can decide to live in residential care communities and other alternative forms of living [34, 35]. In our study, two full-time care nursing homes in Bremen were included.

### Establishment of patient advisory boards and role of patient advocate

Two PABs were set up in two nursing homes in Bremen, Germany, to gain insights into the experiences of nursing home residents made with their personal medication plan and to identify unmet needs. The PAB consisted of volunteering nursing home residents. The findings made during the PABs were planned to subsequently inform the development of a complex intervention for a future clinical trial on polypharmacy together with the study planning team.

A patient advocate (PA) played a crucial role in the conceptualization, organization, and facilitation of the PABs. The role of the PA was created to moderate the PAB meetings and to establish safe and trusting relationships with all study participants, ensuring a deep understanding of their perspectives and encouraging their active engagement in the PABs, as research indicates that older persons may find it challenging to form bonds, potentially leading to reduced contributions [20].

The PA also communicated the findings of the PAB, where nursing home residents could express their interests, desires, and needs, to a study planning team, consisting of a group of researchers, thus, acting as an intermediary between the nursing home residents and researchers. In our study, CH, a researcher with expertise in biometrics, was selected as the PA due to her extensive experience both, in work with older patients and in clinical trial planning.

Nursing home residents were invited to become part of a PABs through purposeful sampling [36]. Potential participants had to be aged 65 or older, residing in one of the two nursing homes that were partaking in our study, prescribed cardiovascular medication, and willing to participate in the PABs, one interview and two group discussions. The use of multiple medications was not an inclusion criterion for the PABs to ensure a broad range of perspectives. Interested nursing home residents were informed about the goals and the function of the PAB and the planned PI interviews and group discussions and received written information about the project as well as a consent form translated in simple language. All information materials for the patients who would later be involved were previously tested. Further, contact details of the PA were provided to offer an additional opportunity to discuss questions and uncertainties during the research process.

Subsequently, interested nursing home residents had a familiarization interview with the PA, as an earlier study identified the familiarization as important [20], and to address any questions or uncertainties. These meetings also provided the PA with information on the patients’ health status and any cognitive, visual, or auditory limitations, which were crucial for planning the PAB meetings. Nursing home residents were provided with basic written information in plain language on clinical research processes and active PPI to prepare for the first PAB meeting.

The study planning team comprised seven researchers, with two additional researchers providing expertise in PPI. Although it is highly desirable for patients to be directly involved as part of the research team throughout the process, health-related restrictions unfortunately prevented us from including members of the PABs as active study team members. However, through regular communication with the two consultants from the target group, we ensured that the patients’ perspectives were represented within the research team.

### General setup of Patient Advisory Boards and Study Planning Team

**Fig. 1:**
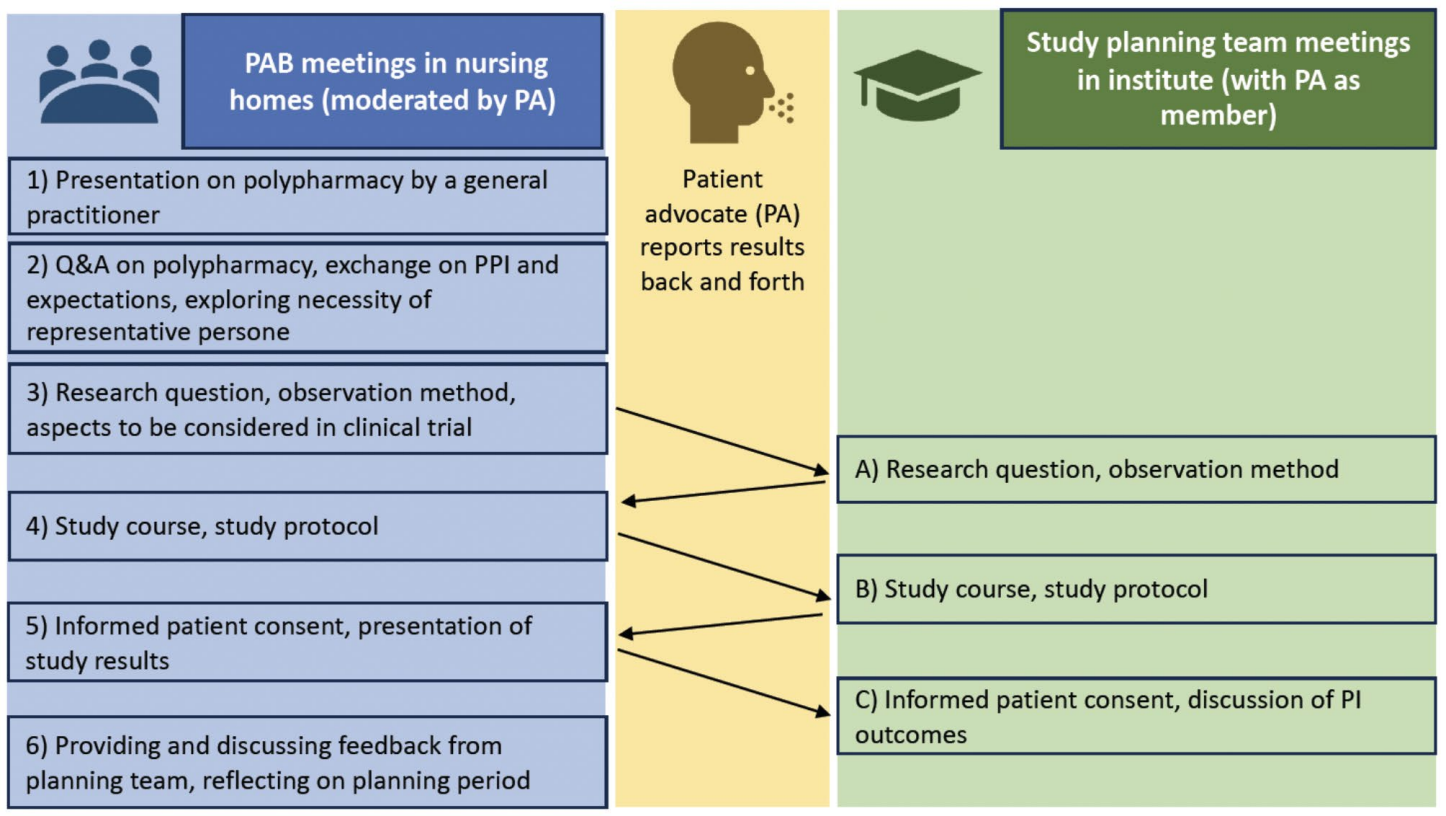
Structure and topics of patient advisory board (PAB), patient advocate (PA) and study planning team

The PABs were established to enable patient involvement in the development of a clinical trial on polypharmacy in nursing home residents. The PABs were held in barrier-free rooms within the nursing homes’ familiar surroundings. Over five months, the clinical trial design was gradually discussed in four PAB meetings (2–5) in each nursing home, along with three study planning team meetings (A, B, C) (see Fig. 1).

On average, PAB meetings lasted approximately 90 min. At the beginning of each meeting, the content of the previous meeting was orally summarized, as preferred by the patients who opted against receiving written minutes beforehand. The PA reported the results from the study planning team and any changes made based on the previous PAB meeting before discussing the next stage of trial design. Participating nursing home residents received a monetary allowance for their participation. Nursing home residents who could not or did not want to attend PAB meetings were invited to individual meetings (*n* = 6) during which the PA and a supporting researcher presented the minutes and main results of the PAB meeting and solicited the nursing home residents’ opinions.

Simultaneously, researchers discussed the same topics of the 3rd, 4th, and 5th PAB in 90-minute meetings moderated by the project coordinator (HJ), within the IPP venue. The study planning team incorporated the results of each PAB meeting into the study design of the clinical trial whenever feasible, with the PA serving as the sole connecting contact.

### Data collection on motivation, expectations, and experiences

Individual interviews (1) and two rounds of group discussions (2 + 3) were conducted before, during and after the project. Additionally, nursing home residents and researchers completed a brief questionnaire at the project’s onset to collect socio-demographic data of all study participants.

#### Interviews for motivation and expectations

Qualitative semi-structured interviews were conducted to capture initial motives and expectations of both nursing home residents and researchers. Thirteen nursing home residents were interviewed in their nursing home rooms by one researcher (KR), with the PA present. Nine researchers (including the PA) were interviewed face-to-face at the university (*n* = 7) or via telephone (*n* = 2). Interview questions were based on motivation theories [37, 38] and relevant PPI research findings [25, 39–42], and the guidelines were finalized through consensus-building among AG, KR, HJ and CH, they were not piloted. On average, patient interviews lasted 18 min (range: 9–36 min.), researcher interviews lasted 16 min (range: 15–36 min.). The interview guide can be found in the supplementary materials S1.

#### Group discussions for experiences and changing expectations

Dual-moderator (CH, KR) guideline-based group discussions were conducted with the thirteen PAB participants (with a separate discussion for each nursing home) and the nine researchers of the study planning team. The discussions aimed to collect experiences and changing expectations of the participants. We chose a group method to facilitate a deeper engagement and discussion of the diverse experiences among participants, allowing for a richer exchange than what could have been achieved through individual interviews [43]. In practice, much of the communication during the discussions with the nursing home residents occurred between the moderators and the participants, rather than among participants themselves. This strong emphasis on moderation was necessary to accommodate the communication styles of our participants, as direct interactions between participants were limited.

Interim group discussions (2) were held midway through the PAB meetings, lasting 60 min for nursing home residents and 35 min for researchers. Final group discussions (3) took place after the final PAB meetings, each lasting approximately 90 min. The final discussion with the study planning team was moderated online by SB. These final discussions assessed original expectations, experiences and gathered advice for future PPI with older patients in clinical research. All group discussion guides can be found in the supplementary materials S2.

All interviews and group discussions were conducted in German, audio-recorded, transcribed verbatim, and de-identified to protect participant identities. The audio recording from the second group discussion with the PAB participants of one nursing home was damaged. However, it was possible to substitute this with very detailed notes taken during the group discussion session. Except for the final group discussion with the study planning team, all group discussions were held in person.

### Data analysis

Thematic analysis was performed collaboratively by researchers SB and IS following Kuckartz’ principles of structured qualitative content analysis [44], with codes generated in MaxQDA Analytics (Versions 2018 & 2020, VERBI GmbH, Berlin, Germany). Both researchers weren’t part of the study planning team but IS advised the PPI. The analysis involved both deductive main categories from the interview guidelines and inductive sub-categories developed during the coding process. All transcripts were coded by SB and IS until consensus was reached.

Comparisons were made between initial motivation and expectations and actual experiences within and between patient and researcher groups. Participants’ personal data were excluded from the analysis and presentation of results. Direct quotes used in this article were translated into English. Descriptive analysis was applied to the questionnaires completed by participants at the beginning of the study.

## Results

All 13 members of the PABs from two nursing homes (NH1: 7 residents; NH2: 6 residents) and all involved researchers (*n* = 9) participated in interviews that explored their motivations, expectations, and experiences. Of the 13 nursing home residents participating in the PAB, eleven were female. The nursing home residents´ ages ranged from 80 to 98 years at recruitment (mean age 87,2), and most had physical and/or cognitive impairments. The researchers were aged between 30 and 53 years (mean age: 40). Table 1 details the characteristics of the involved nursing home residents and researchers.

**Table 1:** Characteristics of involved nursing home residents and researchers

Nursing Home Residents (*N* = 13)			Researchers (*N* = 9)		
**Age**	80–84	5	**Age**	20–34	3
	85–89	3		35–49	3
	90–94	4		50–64	2
	95–99	1		N.N.	1
**Gender**	Female	10	**Gender**	Female	5
	Male	3		Male	4
**Educational level**	Higher	6	**Main task**	Coordination	1
	Secondary	7		Study planning team	6
**Medication/day**	3–4	2		Patient advocate	1
	5->5	10		Support PI	1
	N.N.	1			

### Background of nursing home residents and researchers

Nearly all the residents engaged as nursing home residents were on daily multiple medications, predominantly for coronary heart disease (CHD). Some found the medication burden heavy and sought more information, some explicitly stated their trust in their GP’s management. No nursing home resident had prior clinical trial or research involvement experience. Their understanding of clinical research varied from basic concepts to confusion with patient information. Three researchers had experience with patient involvement in trials, another one expressed that “so far, it has puzzled me as to how I should actually implement it” (F6:9).

Our findings are organized by three major themes: (i) motivation to participate, (ii) expectations of the PAB, and (iii) experiences with the PAB, study planning team and PA. During thematic analysis of all transcripts, fifteen sub-themes emerged within the three major themes that were either unique to one study participant group or similar for both study participant groups (cf. Table 2).

**Table 2:** Sub-themes emerged during content analysis

Major Theme	Study Participant Group	Sub-Themes
*Motivation to Participate*	Nursing Home Residents	• Patient Empowerment and Contribution to Care • Personal Health and Study Interests • Social Interaction and Curiosity
	Researchers	• Advancing Patient-Centered Research through PPI • Compliance with PPI Requirements • Learning about PPI with Older Nursing Home Residents
*Expectations of the PAB*	Nursing Home Residents & Researchers	• Roles and Communication • Topics, Methods and Results
*Experiences with the PAB, Study Planning Team and PA*	Nursing Home Residents	• Empowerment through Involvement in PAB • Understanding Roles and Tasks • Training and Understanding Research • PAB Experiences
	Researchers	• The Role of PA as Mediator • Challenges in PAB Study Planning • Suggestions for Improvement

### Motivation to participate

This chapter summarizes the motivation for participation in this PI project of nursing home residents and researchers, based on the semi-structured individual interviews conducted prior to the first PAB meetings.

#### Nursing home residents’ motivation

To understand the motivation of participating nursing home residents, several sub-themes were identified. These showed that PI was valued as an important addition to research. The social aspect and diversion from daily life were found to be motivating factors, while the nursing home residents were open to most topics for discussion.

#### Patient empowerment and contribution to care

Nursing home residents were motivated by the opportunity to be involved and potentially impact their personal healthcare. One nursing home resident remarked, “it’s good to be involved in what concerns oneself“ (P13:37), highlighting the empowerment that comes from understanding their personal treatments.

However, also a clear desire to contribute to the improvement of other residents’ situations and the enhancement of their respective nursing homes was expressed. Feeling responsible, one nursing home resident stated: “I, uh would like to help ensure that the people who live here may co-create a pleasant aging” (P13:12). Being able to contribute to improvements in the care environment for themselves and for other residents, highlighted a strong sense of responsibility and collective benefit.

#### Personal health and study interests

Some nursing home residents were interested in seeking solutions to personal health concerns or issues related to polypharmacy. This included the optimization of cardiovascular medication, aspirin use and headaches, or medication inaccuracies. Many nursing home residents did not express a specific interest in discussing a particular study topic but were open to any topic that would be offered to discuss. One resident commented, for example: “I let it come to me. And if it’s not for me, I leave. And when I realize that I can contribute something, somehow, or that interests me in particular, […]. I’m open to anything.” (P8:77–79).

#### Social interaction and curiosity

A significant motivator for participation was the desire for interaction and a break from daily routines. Engaging in the study provided a welcome opportunity for intellectual stimulation and social connection. A number of nursing home residents expressed curiosity about the study and were looking forward to a welcome diversion from usual activities.

#### Researchers’ motivation

To understand the motivation of participating researchers, several sub-themes were identified that showed that researchers were in general very interested in addressing PPI in research to make their research meaningful and to meet formal PPI requirements. Working with older patients and developing a study that could subsequently be carried out were also significant motivational factors.

#### Advancing patient-centered research through PPI

Researchers were driven by the progressiveness of PPI in research, aiming to integrate patient perspectives and develop patient-relevant research. Their motivation stemmed from a desire to make research more relevant and impactful for patients. Additionally, some researchers were particularly interested in conducting clinical studies on polypharmacy and saw this as an opportunity to publish findings. This demonstrates a dual motivation to contribute to meaningful scientific knowledge that could be translated into practice and achieve professional recognition through publication.

#### Compliance with PPI requirements

Meeting formal requirements from research funders for PPI and gain hands-on PPI experience was another key motivator. As one researcher noted, “patient involvement is demanded in the applications, but this is mostly just mere lip service” (R5:5). This highlights a desire to move beyond superficial compliance of PPI requirements and involve patient perspectives more meaningfully through a genuine incorporation of the patients’ input into the research.

#### Learning about PPI with older nursing home residents

Researchers were also curious about the feasibility and effectiveness of PABs with older nursing home residents and the potential impact on research outcomes. One researcher expressed particular interest in the practical application of the PA role, indicating curiosity about how involving older nursing home residents could influence the research process and results, and how this could be facilitated by an intermediary role such as the PA. Comparing nursing home residents´ and researchers´ decision influencing factors.

Nursing home residents indicated few reservations about joining the research, some questioned their suitability for the project, with one remarking, “I’ll listen and then it’s up to you to determine if I’m a good fit or not” (P9:10f). Nursing home residents preferred being contacted personally by familiar figures, like nursing home staff, rather than by phone, letter, or email. Positive interactions with researchers, such as appreciating their “nice speech [and] kindness” and not feeling pressured (P4:25), also played a role in nursing home residents’ willingness to participate.

Although researchers were eager to involve older patients, they were concerned about maintaining a “target-oriented” approach due to potential issues with attention, cognition, mood, and communication (R6:20; R7:11). They also worried about consistent participation, given health fluctuations, mobility limitations, and conflicting time schedules.

However, both groups shared a common goal of making research more meaningful and impactful through collaborative involvement.

### Expectations of the PAB

This chapter summarizes the expectations for the PI based on the semi-structured individual interviews conducted with nursing home residents and researchers prior to the start of the PAB meetings.

#### Roles and communication

Nursing home residents looked forward to meeting with other persons, preferring meetings without researchers to avoid academic jargon and promote free expression. However, one nursing home resident hoped to also exchange with researchers at one point during the process. Opinions varied on the effectiveness of individual versus group communication, with some concern that participants might focus more on personal issues rather than on the research. Nursing home residents lacked specific expectations about their roles, “I haven’t thought about that yet“ (P1:116). Many were unclear about their ability to contribute to the research design.

The PA aimed to facilitate open and balanced PAB meetings and to summarize outcomes and queries effectively for the study team without overly filtering nursing home residents’ inquiries so as to not censor their perspectives.

Researchers expected nursing home residents to suggest research topics and ideas for study design, research questions and patient outcomes. They saw the PA as a key intermediary for fostering trust and open communication with the nursing home residents. Researchers held mixed views on direct communication between nursing home residents and researchers. Some saw it as intimidating for nursing home residents, while others viewed it as a “missed opportunity for joined learning” (R5:52). Meeting nursing home residents in person was seen as beneficial for understanding different needs and perspectives by some researchers, and one anticipated that directly talking with researchers would be more empowering for nursing home residents. Providing feedback to nursing home residents on the use of their input was seen as crucial.

#### Topics, methods and results

Few nursing home residents expressed interest in particular research topics, such as patient-doctor communication, polypharmacy, medication information, and loneliness. However, most did not have specific topics for discussion and were simply curious about the process. Nursing home residents trusted the organizational process and did not have specific expectations for the PAB but hoped for tangible results.

Researchers were particularly interested in topics related to polypharmacy, including its causes and medication optimization. Researchers recognized that PPI is underutilized in Germany, aiming to understand PABs’ and PAs’ potential and receive guidance on integrating PPI in research. However, some researchers held modest expectations regarding patient involvement, focusing more on including patient perspectives than anticipating substantial contributions to research. Concerns about the feasibility of implementing nursing home residents’ suggestions were raised. One researcher worried that these suggestions might either be overlooked or prove unfeasible. Echoing this sentiment, another researcher suggested that embracing PABs’ recommendations might require researchers to reevaluate their academic practices.

### Experiences with the PAB, study planning team and PA

This chapter details experiences related to PI through PABs, drawing from interim and final group discussions of both PAB members (nursing home residents) and the study planning team (researchers).

Nursing home residents reported experiences related to the effects of PAB, understanding roles and tasks, training and introduction, and PAB conduct.

#### Empowerment through involvement in PAB

Nursing home residents valued their role in the PABs, as it gave them a sense of agency and recognition, trusting that sharing knowledge and experiences among diverse groups might increase the chances of altering medication strategies. They felt respected and heard. Involvement in PABs was linked to mental acuity and open-mindedness. This involvement provided valuable experiences, enhancing nursing home residents’ awareness of the subject and increased their confidence in discussing medication management with their physicians.

#### Understanding roles and tasks

Nursing home residents initially had a limited understanding of their roles within the PAB, but over time, they grew to see themselves as important contributors by representing the perspective of older patients and providing important information. From their perspective, future clinical research should involve nursing home residents. However, some nursing home residents cautioned that not everyone may be suitable for PAB participation due to the complexity of the discussion, which could lead to confusion or to too much “tohuwabohu” (chaos) in the research process (final group discussion P).

#### Training and understanding research

While nursing home residents were given an introduction to the PAB process and the topic of polypharmacy by a physician researcher (Fig. 1, Meeting 1), they expressed a need for more training, particularly to better understand medical terminology and the topic of polypharmacy, as well as the research concepts, including their role and involvement in the process. One nursing home resident stated in the final group discussion that managing multiple medications involves complicated information that can be difficult to understand, especially when explained with medical (Latin) terms. Opinions varied regarding the content and effectiveness of such training, with a preference for lecture-style over written formats to aid comprehension.

#### PAB experiences

PAB meetings were seen positively, providing a structured platform for discussion. Nursing home residents appreciated both group and individual interactions, noting differences in the openness of communication in each setting. They also appreciated structured, organized meetings and felt the group size was appropriate. Participation was made convenient by providing transportation to the meeting location. However, there was uncertainty about how PAB outcomes would be applied and whether they could benefit others.

Nursing home residents expressed a desire for continued involvement. In their final group discussions, nursing home residents compared their expectations with actual experiences. Met expectations included reduced medication intake, a break from daily routines, and learning new things, such as the importance of reporting concerns. However, expectations like improved care for older patients and personal health benefits, such as less pain, were not met.

Researchers reported experiences related to the use of PA as mediator, challenges in PAB study planning, and suggestions to improvement of PABs.

#### The role of PA as mediator

The PA was valued as a mediator and intermediary between nursing home residents and researchers. The PA’s involvement was believed to foster trust with nursing home residents, evidenced by continued communication, such as nursing home residents emailing the PA about personal matters like vacations. The PA’s presence encouraged nursing home residents to express opinions and concerns openly, helping to balance power dynamics and hierarchical structures. However, the PA noted that patience and time were crucial for the success of PABs.

#### Challenges in PAB study planning

Researchers valued the concept of PPI and PABs but faced challenges involving older nursing home residents. They noted that the complexity of the polypharmacy topic hindered understanding, rather than the age of participants. One researcher commented, “It’s not just with nursing home residents that it is difficult to be very specific about the topic of medication and evaluating what prioritization and my goals are, but I also find this to be the case with much younger people, at least, because it is simply such an incredibly complex topic […].” (final group discussion R). A need for a more realistic approach to PPI in research proposals was highlighted, considering patients’ abilities and interests.

The PAB’s influence led to the decision not to conduct a planned polypharmacy study, which the researchers considered a good thing: “I think it was definitely very good that we didn’t just do the study as we would have experienced failure - and the reason we didn’t just do the study was actually the patient advisory board. […] I would put it this way, they didn’t explicitly tell us: ´Pay attention. What you’re imagining with the people that are here, that doesn’t work´ - but they basically communicated it more or less indirectly” (final group discussion R).

One researcher suggested a more realistic approach in PPI research proposals, emphasizing genuine involvement based on nursing home residents’ abilities and interests, not just fulfilling a requirement.

A personal introduction to PAB members may have been beneficial for building confidence and understanding the group dynamic. Researchers debated the necessity of direct contact to the nursing home residents, which in their opinion may depend on the study topic and the researcher’s familiarity with the field.

#### Suggestions for improvement

Researchers proposed several strategies to improve PAB effectiveness:

- *Divide PAB* in two groups based on cognitive abilities, with support for those with limitations.
- *Incorporate Additional Board* of next-of-kin or medical staff for diverse perspectives.
- *Use Case Vignettes* for focused PAB discussions.
- *Tailor Content* to suit participant groups.
- *Honor Interview Preferences* for individual or group interviews.
- *Encourage Researcher Observations* of PAB meetings for deeper understanding.

Despite not leading to a specific study design, the PAB’s role in research, especially with older nursing home residents, was deemed essential, highlighting the need for methodological adjustments based on participant needs.

## Discussion

This research primarily aimed to explore the motivations, expectations, and experiences of involving older nursing home residents, specifically nursing home residents, in PPI for a clinical trial on polypharmacy. The focus on nursing home residents was driven by their underrepresentation in research and the unique insights they could offer due to their specific healthcare needs and living circumstances. The study involved PABs in two nursing homes in Bremen, Germany, moderated by a PA, who facilitated communication between the PABs and the research team. Data were collected through semi-structured interviews and group discussions with both nursing home residents and researchers.

### Discussion of main findings

This study highlights varied motivations and expectations in PPI and the complexities of engaging nursing home residents in PABs. It reaffirms the growing recognition of PPI’s value in enhancing health research relevance and quality [45]. However, it also points out the unique challenges and opportunities in engaging a specific patient group, here older nursing home residents, in meaningful research involvement.

#### Varied Motivations and Expectations

There is a divergence between nursing home residents’ and researchers’ motivations and expectations. While researchers’ predominant focus was to consider nursing home residents’ perspectives in the design of a study related to polypharmacy and to learn about the methods of PPI, nursing home residents appreciated the social interaction, the diversion from everyday activities, the focus on personal health related issues and the general notion of “being heard”. Nursing home residents’ expectations primarily concerned organizational aspects of PABs, such as timing and group setup, rather than contents. They had limited initial understanding of their roles in PPI.

#### Experiences with PABs and the role of PA

Nursing home residents’ experiences with PABs were largely positive. They appreciated the mental stimulation and the opportunity to discuss their health needs, despite some confusion about their roles. They reported benefits from the involvement, such as valuable experiences, enhanced awareness of the subject and confidence to discuss own medication management with their GPs. However, the complexity of the study topic, polypharmacy, appeared challenging for nursing home residents. Questions about medication were sometimes deflected or vaguely answered, suggesting difficulty in understanding. These challenges may stem from cognitive issues, low health literacy, or lack of interest. Most importantly, it shows that the way in which the topic was addressed needs be reconsidered. Although many nursing home residents expressed to feel more comfortable in having the PAB without the presence of researchers, some appeared to have had some interest in meeting the researchers. However, it is left unclear if this interest was related to the wish for a short introductory meeting or to have study related face-to-face conversation with the researchers. Schilling et al. report in their study on a patient board with patients aged 20–65 years, that patients would have appreciated a closer and more frequent involvement and contact with the researchers, while the researchers felt that the level of involvement was adequate [26]. This highlights the divergent views regarding the desired level and frequency of involvement, suggesting that enhanced communication could lead to better management of expectations.

Researchers recognized the moderated PAB approach, including a PA acting as mediator, as effective in creating a trusting and open environment for diverse perspectives. This underscores the importance of having dedicated individuals who can bridge the gap between the technical language of research and the everyday experiences of nursing home residents. However, the need for time and patience in conducting PABs with older nursing home residents was also evident, suggesting that PPI in such contexts requires a long-term commitment and understanding of the PAB participants’ pace and needs.

#### Challenges in PPI with older patients

Our study underscores the challenge of involving nursing home residents in PPI. The complexity of the study topic, particularly polypharmacy, posed significant comprehension difficulties for older nursing home residents. This finding suggests a need for more tailored PPI approaches that consider the cognitive and sensory capabilities of older persons. It also highlights the importance of simplifying research topics and methods to ensure meaningful participation. This is in line with the challenges identified in a review of nine studies on PPI of older patients. Most studies proposed visual or auditive aids and accessible material (e.g. plain language, large fonts) to enhance communication and focus, and organizational and planning facilitators to take into account mobility and time availability such as early planning of PPI and setting [20].

#### Methodological adjustments for effective PPI

Ideas for methodological adjustments include dividing PABs based on cognitive abilities, involving additional boards for diverse perspectives, using case vignettes to stimulate directed group discussions on realistic situations – such as adding a medication to lower the blood pressure that might increase the risk of falls – rather than having more general exchanges, tailoring content, considering interview preferences, and encouraging direct contact between researchers and patients. Such adjustments can enhance the feasibility and effectiveness of PPI and have been reported by studies on PPI with older patients before [17]. Further methods need to be discussed on how to best provide the participating patients with a deeper understanding of their role within and the impact of the PAB output, managing expectations beforehand regarding the contents and outputs of the PAB. Schilling and Gerhardus identified seven areas of challenges in their literature review when involving people with old-age-related conditions: diversity, communication, location, relationship, timing, continuity and support [20]. While some challenges relevant for PPI (recruitment, diversity and equity) were characterized as being non-specific to methodological approaches, others were related to specific methods [20]. Interestingly, none of the studies included in the review reported on PPI training for researchers. Baldwin et al. found that several studies emphasized the need to evaluate the PPI process to optimize its approach and increase its value for performing research [17].

#### Realistic approaches to PPI

Our findings reveal a gap between the idealistic goals of PPI and the practical realities of implementing it, especially with older nursing home residents, highlighting the need to adopt more realistic and flexible approaches to PPI, considering the specific needs and abilities of the target group. Also, some studies involving “younger” patients suggest that training and frequent involvement are highly appreciated. Schilling et al. (2019) report about experiences of a patient board involving patients between 20 and 65 years where patients expressed their wish for further clarifying their roles and tasks while researchers found it challenging to incorporate PPI within the methodological approach [26]. Nonetheless, researchers and patients reflected in hindsight that PPI during the trial design stage may have been beneficial [26]. Meaningful, non-suggestive tasks that actually influenced the study conduct rather than tokenism were also mentioned as challenging within some studies involving older persons [46, 47]. Within our study, the PAB insights revealed that the planned polypharmacy study did not seem feasible and appropriate to be planned with the target group. Therefore, in a future project PPI should include next-of-kin and medical staff as suggested by the researchers (see results) to facilitate the conduct of the planned study. This learning underscored a crucial lesson: the most profound outcome of PPI can sometimes be the recognition of what is unfeasible or unsuitable.

### Strengths and limitations

This study provides insights into the motivations, expectations, and experiences of older nursing home residents in PPI, an area that has been relatively underexplored [30] as most PPI studies with older patients focused on the involvement of patients with dementia [20].

However, the study had some limitations: This study involved only patients (nursing home residents) in the PAB, possibly overlooking other valuable experiences and perspectives pertinent to the topic. Not including insights from nursing home staff, next-of-kin, or guardians may limit the amplitude of understanding regarding patient involvement in the context of care of older patients.

The study did not investigate reasons for non-participation of patients or their next-of-kin. Existing studies indicate the factors contributing to non-participation can vary, encompassing insufficient information, lack of recognition of personal resource limitations or health-related concerns [48]. Understanding these factors would add to a comprehensive analysis of PPI in this specific setting of nursing homes.

Due to the COVID-19 pandemic, the conduct and analysis of the study was postponed. These delays meant that patients that were interested in getting involved as co-authors, were no longer available to participate in the conceptualization or the writing of this paper.

The fact that some of the authors had double functions could be perceived as a limitation. For instance, KR not only supported the meetings of the PABs but was also involved in data collection. Further, IS was involved as PPI advisor and conducted data analysis jointly with SB, who was further not involved with the patients or researchers. While this arrangement on the one hand enabled a deeper understanding of the PABs, it might on the other hand have affected the objectivity of data collection and analysis.

## Conclusion

This study suggests that patient involvement in the design of a study is highly sensitive to the target group involved. Involving nursing home residents of high age through the concept of PAB will need close support in the provision of information on their role, (PAB or individual interview) structures that enable them to openly voice their opinions, and content adapted to align with their cognitive and experience-based abilities. Researchers need to be mindful of potential barriers surrounding patients’ needs and (cognitive) abilities when preparing and structuring the PAB meetings or other PPI formats.

In conclusion, our study contributes valuable insights into the practical implementation of PPI in clinical research design development with nursing home residents. It highlights the need for a more nuanced, patient-centric approach that recognizes the distinctive challenges and contributions of this target group. With an ageing population, these insights will become increasingly critical in shaping research that not only upholds scientific rigor but also demonstrates social relevance and impact.

## Electronic supplementary material

Below is the link to the electronic supplementary material.

Supplementary Material 1

Supplementary Material 2

## Data Availability

The datasets generated and analyzed during the current study are not publicly available due to data protection regulations. This is part of the guarantee the patients and researchers received before they gave informed consent.
