# Supplementary material for: Engaging nursing home residents in clinical research: insights from a patient advisory board, a patient advocate, and a study team
Source: Res Involv Engagem. 2024 Oct 28;10:111. doi: 10.1186/s40900-024-00648-1 (PMC11514759; doi:10.1186/s40900-024-00648-1)
Supplement: Supplementary file 1 — Supplementary Material 1 [file 40900_2024_648_MOESM1_ESM.pdf]

Guide for Individual Interviews with Nursing Home Residents

Introduction to the Interview

- Explain the purpose of the project and how the interview will proceed.
- Check if there are any questions before starting.
- Obtain consent for audio recording.
- At the beginning of the recording, state the location, date, and attendees.

Questions for Nursing Home Residents

1. Do you know what a clinical trial is?
   - If yes: Have you had any experience with clinical trials? What kind?
   - Were you actively involved in the study? How? Did you have a say in the clinical trial (e.g., in planning or execution)?
   - If no/I'm not sure: Explanation of what a clinical trial is.
2. Why do you want to participate in this project/on the patient advisory board?
   - What influenced this decision?
   - What convinced you?
   - What doubts did you have?
   - What did you find challenging?
   - What spoke against it?
   - Why do you think other older people do not participate in a patient advisory board?
3. Was the contact made with you satisfactory, or could we improve it? How?
   - What aspects are important to you in the initial contact?
4. Did you receive enough information from us? Was anything missing? Do you feel well-prepared for the upcoming discussion on the patient advisory board?
5. What do you expect from your participation in the project/on the patient advisory board?
   - What are your goals for the patient advisory board? What benefits do you think the board can provide?
   - How should the board be structured to achieve these goals? What aspects are important to you in the design of the board?
6. How do you envision working with the other patients and researchers? (Role, tasks, communication...)
   - Are there topics or tasks you are particularly looking forward to? Or things you are unsure about?
   - Is there a specific topic or task in the project that you are excited about?
7. How could the involvement of (older) patients in research be improved?
8. Is there anything else you would like to add? Anything else you'd like to share with us?

Thank you!

Guide for Individual Interviews with Researchers

Introduction to the Interview

- Explain the purpose of the project and how the interview will proceed.
- Check if there are any questions before starting.
- Obtain consent for audio recording.
- At the beginning of the recording, state the location, date, and attendees.

Questions for Researchers

1. Do you have experience with the active involvement of patients in clinical trials? If yes, what kind?
2. What motivated you to support our project?
    - What factors influenced this decision?
    - What challenges did you encounter?
3. How do the requirements of your study's funder (BMBF) and possibly your employer play a role for you? What impact do planned publications have?
4. What are your expectations for a Patient Advisory Board (PAB)?
    - What could be motivating or discouraging for others considering participation in a PAB?
5. What are your goals for the PAB? What benefits do you think the PAB can provide?
    - How should the PAB be structured to achieve these goals? What aspects are important to you in its design? What fosters an effective PAB?
6. What are your expectations for the study planning team?
    - What could be motivating or discouraging for others considering participation in a study planning team?
7. What are your goals for the study planning team? What benefits do you think the study planning team can provide?
    - How should the study planning team be structured to achieve these goals? What aspects are important to you in its design? What fosters an effective study planning team?
8. How do you envision the collaboration between patients and researchers in our project? (Role, tasks, communication...)
9. What role do you believe the Patient Advocate plays?
    - What are their responsibilities?
10. What topics or tasks are you particularly excited about?
11. What challenges do you foresee regarding the PAB/study planning team?
    - What expectations might be placed on you by others?
12. Where do you see the need for improvement in the active involvement of patients in clinical trials?
    - What opportunities do you see for enhancing active patient involvement?
13. Are there any other points that are important to you or that you would like to add?

Thank you!
