# Supplementary material for: Engaging nursing home residents in clinical research: insights from a patient advisory board, a patient advocate, and a study team
Source: Res Involv Engagem. 2024 Oct 28;10:111. doi: 10.1186/s40900-024-00648-1 (PMC11514759; doi:10.1186/s40900-024-00648-1)
Supplement: Supplementary file 2 — Supplementary Material 2 [file 40900_2024_648_MOESM2_ESM.pdf]

Guide for Interims Group Discussions with Nursing Home Residents

Opening of the Group Discussion

- Explanation of the topic and structure of today's group discussion (moderation, openness, audio recording, letting others speak, etc., cf. U. Flick)
- Emphasize that the questions are intended to provide us with a progress update
- Clarify any uncertainties or questions
- Request consent for audio recording
- State the location, date, and participants at the beginning

Questions Regarding Patient Involvement

1. What experiences have you had with the PAB so far?

- What do you particularly like?
- What can be improved?
- What would you keep the same, and what would you change?
- How did you feel about the group size?
- What can others learn from your experiences?

2. How much effort is involved in attending the PAB meetings?

- Should we have met at different intervals (more or less frequently)?

3. Has your participation in the PAB had any effect on you or changed anything?

- Has your attitude towards the doctor-patient relationship changed? If so, how?
- Has your attitude towards medication changed?

4. Is it useful to organize a joint meeting with researchers and nursing home residents?

Questions Regarding Study Planning

5. How should informational materials for nursing home residents/patient involvement be designed (e.g., written materials: simple language, images, page count, or perhaps video/poster)?

6. What do you wish for the next PAB meetings? What can we improve for the next meetings?

7. Are there any other points you consider important or would like to add regarding a clinical study on the topic of "Optimization of medication for nursing home residents"?

Thank you!

Guide for Final Group Discussions with Nursing Home Residents: Reflection

Welcome the participants

- Announce the topic and procedure of the group discussion
- Obtain consent for audio recording and analysis
- Mention the location, date, and attendees at the beginning

Questions for Discussion

1. How did you perceive the group discussions (group dynamics, etc.)?

- Did you feel accepted and appreciated by the other residents?
- Did you feel you were treated with respect?
- Was the duration of the meetings (approximately 1.5 hours) appropriate?
- Were the intervals between meetings acceptable?
- Did you find the gatherings exhausting (e.g., getting to the room, the organization, etc.)?
- How did you feel when you were directly addressed by name and asked for your opinion?

2. How did you perceive the individual conversations?

- Do you think you were able to open up more in one-on-one conversations?
- Was it more comfortable for you when the Patient Advocate visited you in your apartment/room?

3. We discussed the following topics: XYZ. Which topic did you find particularly exciting/interesting? What was especially important to you?

4. At the beginning of the project, you had the following expectations: XYZ. Would you say your expectations were met?

5. How did you perceive your participation in the project?

- Would you participate in this project again?
- If you were to participate again, would you prefer group discussions or individual conversations?

6. Is there anything that stands out to you as particularly positive or negative from the project or the discussions?

7. Were you able to take anything personally from the PAB meetings?

8. In your opinion, what could we do better if we were to organize another discussion round with older patients/ nursing home residents?

- Do you have any advice for the researchers?

Optional: 9. Could you briefly summarize what your role in the project was?

Thank you!

Guide for Interims Group Discussion with Researchers

Opening of the Group Discussion

- Location and date
- Topic: “Study planning with patient involvement”
- Duration: approximately 1 hour
- Consent for audio recording

Questions

1. How do you perceive the collaboration within the study planning team so far?

- What do you particularly like?
- What can be improved?
- What would you keep the same, and what would you change?

2. How do you think the collaboration between researchers and nursing home residents could be further improved?

3. What are your thoughts on how to better integrate the needs of nursing home residents into the study planning process?

4. Why might it be beneficial to organize a joint meeting between researchers and nursing home residents/patients?

5. How is the concept of the Patient Advocate working so far?

- Are you satisfied with everything?
- Do you have any comments or suggestions for changes (e.g., regarding individual conversations)?

6. Which expectations regarding the collaboration with nursing home residents through the advocate have been met, and which have not?

7. What do you expect from a clinical study on the topic of “Optimization of medication for nursing home residents”?

8. Are there any other points you consider important or would like to add regarding a clinical study on the topic of “Optimization of medication for nursing home residents”?

Thank you!

Guide for Final Group Discussion with Researchers

Opening of the Group Discussion

- Location and date
- Topic: "Study Planning with Patient Involvement"
- Duration: Approximately 1 hour
- Consent to audio recording

Questions

1. The results of the individual interviews regarding your expectations showed... (anonymous summary of results). How do you see things now?
2. How did you find the collaboration within the study planning team?
    - What went particularly well? (What would you keep?)
    - What could be improved? (What would you change?)
3. How do you think the collaboration could be improved:
    - Between researchers?
    - Between researchers and nursing home residents/patients?
4. Were any relevant people missing from the advisory board/study planning team?
5. Would it be useful to organize a joint meeting with researchers and nursing home residents/patients?
6. Did the project meet your expectations? Were there any disappointments? What were you not satisfied with?
7. Which expectations for working with nursing home residents/patients through the Patient Advocate were met and which were not? What challenges did you encounter? Would you have preferred direct collaboration with the nursing home residents?
8. How do you think the needs of the nursing home residents could be (better) implemented into the study planning?
9. Were nursing home residents the right target group?
10. What are your recommendations for planning future involvement?
11. Under what circumstances would you participate in a study planning team again?
12. If we revisit the goals of INVOLVE: Have we achieved them? What is missing?

Goals of INVOLVE:

13. [What do you expect from a clinical study on the topic of optimizing medication for nursing home residents?]

- Are there any other points regarding a clinical study on the topic of "optimizing medication for nursing home residents" that are important to you or that you would like to add? What have we learned?

Thank you!
